# Cytokine Profile As a Marker of Cell Damage and Immune Dysfunction after Spinal Cord Injury

**DOI:** 10.32607/actanaturae.11096

**Published:** 2020

**Authors:** G. B. Telegin, A. S. Chernov, N. A. Konovalov, A. A. Belogurov, I. P. Balmasova, A. G. Gabibov

**Affiliations:** Branch of Shemyakin and Ovchinnikov Institute of Bioorganic Chemistry Russian Academy of Sciences, Pushchino, 142290 Russia; N.N. Burdenko National Scientific and Practical Center for Neurosurgery, RF Health Ministry, Moscow, 125047 Russia; Shemyakin and Ovchinnikov Institute of Bioorganic Chemistry Russian Academy of Sciences, Moscow, 117997 Russia; Evdokimov Moscow State University of Medicine and Dentistry of Russia’s Ministry of Health, Moscow, 127473 Russia

**Keywords:** spinal cord injury, cytokines, cellular response

## Abstract

This study reviews the findings of recent experiments designed to investigate the cytokine profile after a spinal cord injury. The role played by key cytokines in eliciting the cellular response to trauma was assessed. The results of the specific immunopathogenetic interaction between the nervous and immune systems in the immediate and chronic post-traumatic periods are summarized. It was demonstrated that it is reasonable to use the step-by-step approach to the assessment of the cytokine profile after a spinal cord injury and take into account the combination of the pathogenetic and protective components in implementing the regulatory effects of individual cytokines and their integration into the regenerative processes in the injured spinal cord. This allows one to rationally organize treatment and develop novel drugs.

## INTRODUCTION

Spinal cord injury (SCI) is a serious global health problem which often leads to severe lifelong disability [1, 2]. According to the WHO, up to 500,000 people, including young patients aged 20–35 years, suffer from SCI annually in the world [3].

Broad opportunities for studying the morphological and pathophysiological changes in patients with SCI, which are necessary for developing rational treatment strategies, have made it possible to progress from clinical observations to developing experimental models [4]. This approach has allowed one to elucidate many pathogenetically significant mechanisms that underly the development of this pathology, including those associated with the immune responses to the injury; so, these responses were classified into immediate and chronic post-traumatic reactions [5].

## 1. IMMUNE AND CYTOKINE RESPONSES DURING THE ACUTE POST-TRAUMATIC PHASE AFTER A SPINAL CORD INJURY

Two different phases are distinguished in the pathogenesis of the immediate post-traumatic period of spinal cord injury. Each of them leads to a complex of pathophysiological reactions in response to the damage to the nervous system [6, 7].

The first post-traumatic phase that starts on the first day after trauma exposure involves the damage mechanisms and disorders associated with it. Neurons, astrocytes, oligodendrocytes, and other components of nerve signal transmission are physically affected, which is accompanied by disorders in vascular components, including the blood–brain barrier (BBB) [8, 9, 10]. This results in tissue infiltration by inflammatory cells [11, 12, 13].

The second post-traumatic phase involves the endogenously induced degradation of the nervous tissue and associated consequences [14]. Increased glutamate level in the damaged spinal cord (SC) tissue causes neuronal excitotoxicity, a pathological process leading to neurotransmitter-mediated damage and death of nerve cells, due to the excess of intracellular Ca^2+^. This promotes the accumulation of reactive oxygen species [15, 16, 17], which, in turn, damage cellular components, such as nucleic acids, proteins, and phospholipids, and cause significant cell loss and subsequent neurological dysfunction [18, 19].

The inflammatory response to primary structural changes in the spinal cord is accompanied by the release of a large number of regulatory peptides, including proinflammatory ones, and cytokines [20, 21]. Cytokines are synthesized by activated macro- and microglia, damaged vascular endothelium, as well as the immune system cells mobilized from the systemic circulation to the injury site and the adjacent areas, due to changes in the BBB permeability [22].

**Fig. 1:**
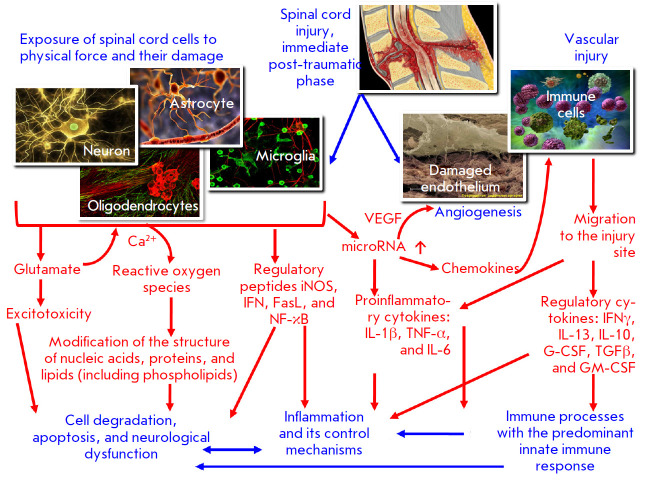
The pathogenetic mechanisms of the immediate post-traumatic phase after SCI that trigger the innate immune system response.

*Figure 1* shows the main pathogenetic mechanisms involved in the immediate post-traumatic phase of SCI, as well as the general role played by immune system cells and cytokines in its development.

It was found that a series of immunologically significant molecules, including tumor necrosis factor (TNF-α), inducible nitric oxide synthase (iNOS), nuclear factor (NF)-kB, interleukin (IL)-1β, and/or a factor of the apoptosis Fas ligand (FasL), are activated as early as within a few minutes after SCI [23, 24, 25]. Activation of these molecules further results in inflammation and other forms of important neurological disorders [14].

Activated astrocytes are the main source of all these factors: they account for about 30% of all cellular components; overexpression of the microRNA miR-136-5p in these cells during SCI is one of the inducers of proinflammatory factors and chemokines (primarily TNF-α and IL-1β) [26, 27, 28]. This process triggers an inflammatory immune response involving type 17 T-helpers [29]. Angiogenesis is another concomitant effect of SCI mediated by microRNA (miR-210) [30, 31].

It should be emphasized that it is the endogenous cells (neurons and glial cells) of the human spinal cord but not white blood cells that contribute to the early production of IL-1β, IL-6, and TNF-α in the post-traumatic inflammatory response [32, 33, 34].

However, one should not underestimate the role played by immune cells as a source of proinflammatory cytokines in a spinal cord injury. This is facilitated by hemorrhage in the spinal cord tissue after damage to it [35, 36], which enables infiltration of the affected areas by neutrophils, monocytes/macrophages, and T cells [37-40] (i.e., cells releasing the same factors TNF-α, IL-1α, IL-1β, and IL-6) [41, 42].

In general, these cytokines reach their peak level 6–12 h after the injury; they also induce an inflammatory response in acute and subacute periods and expand the lesion in the rostral and caudal directions [43, 44, 45]. Activated microglia and macrophages infiltrating the spinal cord have been shown to be responsible for the subsequent necrosis and apoptosis of neurons, astrocytes, and oligodendrocytes at the injury site [46, 47], thus worsening the neurological outcome [48, 49].

As for the signals of cytokine release, they can enter the cells through the Toll-like receptors (TLRs) of the spinal cord [50, 51]. TLRs are best known as the structures for pathogen recognition and initiation of the innate immune response [52, 53]. However, they can also detect tissue damage and trigger sterile inflammation by binding to endogenous ligands typical of stressed or damaged cells. In addition to the cells associated with the immune system, TLRs have also been revealed in the neurons of the central nervous system (CNS) and glial components, including microglia, astrocytes, and oligodendrocytes [54, 55]. Considering the above, Toll-like receptors can play both a direct and indirect role in a spinal cord injury [56]. The indirect effects are most likely mediated by microglia or the immune cells penetrating the damaged CNS tissue [57]. It was also revealed that the restorative responses in ischemic disorders after a spinal cord injury occur with predominant involvement of Toll-like receptor 3 and subsequent regulation by TLR4 [58].

Modulation of proinflammatory and immune effects in the spinal cord tissue during injury occurs with the involvement of interferons due to the increased concentration of stimulators of the interferon genes (STING) in the tissue [59, 60].

Another immunological effect is observed during the first 24 h after the spinal cord injury: the number of natural killer (NK) cells with an activated phenotype increases significantly, manifesting itself as overexpression of CD69, HLA-DR, NKG2D, and NKp30 on their membrane, as well as enhanced cytotoxic activity [61]. Furthermore, an increased level of the brain-derived neurotrophic factor (BDNF), which can be secreted by vascular endothelial cells, was found in patients’ plasma samples, which strongly correlated with the percentage of NK cells and the level of activation molecules CD69 and NKp30 on their surface during this phase after SCI. [62].

Early intervention to reduce inflammation and prevent apoptosis has long been a strategy in treating spinal cord injury. However, the growing body of knowledge in this field suggests that the inflammatory process has apparent protection aspects that should not be ignored during therapy [63].

One of the mechanisms of innate immune defense during inflammatory response after a spinal cord injury is associated with the unique role played by mast cells [64]. Mast cells are abundant in the CNS and play a rather complex role in the development of neuroinflammatory disorders. In particular, astrogliosis and infiltration of T cells increase in mast-cell-deficient mice, while functional recovery after a spinal cord injury is significantly reduced in these animals [65]. Moreover, these mice have significantly increased levels of cytokines MCP-1, NFα, IL-10, and IL-13 in the spinal cord. Data have been obtained on the relationship between these phenomena and the fact that, at an equal number and functional activity of mast cells, their chymases cleave MCP-1, IL-6, and IL-13, thus indicating the protective role played by these cellular elements in the development of inflammatory changes in the nervous tissue during a spinal cord injury [66].

The pattern of cytokine and hormone secretion after spinal cord injury largely depends not only on the mechanisms of induction and immune response, but also on injury severity. For instance, experiments in a rat model clearly demonstrated similar differences in the secretion of the vascular endothelial growth factor (VEGF), leptin, interferon-γ-induced chemokine IP- 10, IL-10, IL-18, the granulocyte colony-stimulating factor (G-CSF), and chemokine fractalkine in animals’ plasma. In contrast to the thoracic spine trauma, injury to the cervical spine is accompanied by a reduced expression of these mediators; this is probably due to sympathetic dysregulation, which is associated with higher injury severity [67, 68]. Experiments on mice have also demonstrated that the involvement of the cytokine profile in the systemic changes of interleukins such as IL-3, IL-6, IL-10, IL-13, and G-CSF after a spinal cord injury to the lower thoracic region (Th910) is accompanied by the activation of T lymphocytes and neutrophils during the immediate post-traumatic phase of the observed changes [69].

It should be noted that, in addition to astrocytes and microglia, IL-10 is also produced by macrophages, B cells, and Th2 cells [70, 71]. Being an immunomodulator, IL-10 stimulates the formation of regulatory T cells, while suppressing the activity of Th1 and NK cells [72].

**Fig. 2:**
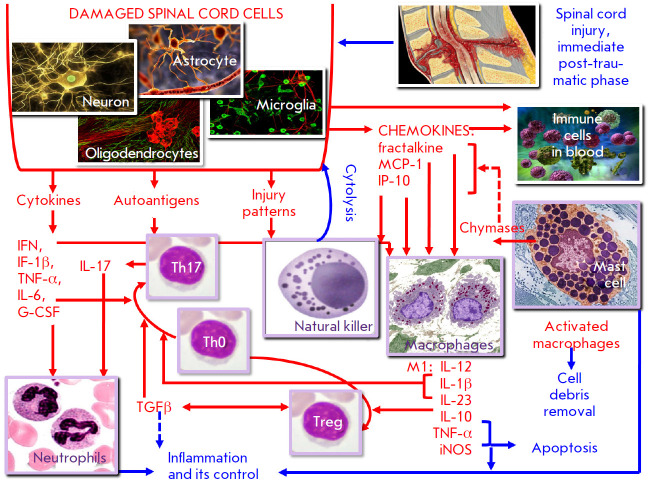
Specific characteristics of the immune response in the immediate post-traumatic phase after SCI

Thus, the immunopathogenetic mechanisms primarily associated with innate immune cells and predominantly proinflammatory cytokines are induced during the immediate post-traumatic phase after a spinal cord injury. *Figure 2* is an attempt to summarize the linking mechanisms of these pathogenetically significant immune responses to the spinal cord injury described in modern publications. The following information regarding the interaction between immunocytes can be added to the scheme.

Damaged neurons and neuroglial cells after a spinal cord injury are a source of chemokines (fractalkine, MCP-1, and IP-10) [67, 69] that target monocytes/ macrophages, as well as lymphocytes and promote their entry into the lesion site. Mast cells are one of the first cells (among the innate immune cells) to exert an effect on the injury site. As already mentioned, mast cells can regulate chemokine secretion; however, their role is far from clear. On the one hand, these cells can be a source of cytokines and other mediators that promote inflammation [73]. On the other hand, chymases released from mast cells during their activation and subsequent degranulation can destroy chemokines and proinflammatory cytokines, thus limiting the intensity of the inflammatory responses [66].

Most chemokines produced by the cells of an injured spinal cord promote the recruitment of monocytes/ macrophages [74], which eliminate cell debris, while chemokine IP-10 also recruits NK cells [75]. The involvement of NK cells in the innate immune response is also facilitated by the fact that spinal cord cells express injury patterns in trauma. These, in particular, include stress-induced molecules (MICA, MICB), which are ligands for NKG2D receptors [76]. In turn, they are overexpressed by NK cells in a spinal cord injury [60]. At first glance, manifestations of the cytotoxic activity of NK cells against the nervous tissue in a spinal cord injury significantly aggravate the destructive processes during trauma [60]. However, the involvement of NK cells in the elimination of exclusively cells carrying injury patterns contributes to a more rapid suppression of destructive processes at the site of a spinal cord lesion.

This study, focused on another crucial player, macrophages, under conditions of tissue damage has demonstrated that their activation involves two stages. During the first stage, these cells acquire an inflammatory (M1) phenotype, which is mediated by endogenous molecules released during cellular damage. At later stages, when reparative processes are triggered in response to damage, the activated macrophages are polarized into the resident (M2) phenotype [77]. In this regard, one can assume that M1 macrophages are predominantly produced during the immediate post-traumatic phase of spinal cord injury. Their induction is also mediated by interferons [78], which accumulate, as has already been reported, in damaged tissues during a spinal cord injury [59]. These macrophages secrete IL- 12, IL-10, IL-1β, IL-6, IL-23, IL-21, TNF-α, and iNOS, characteristic of this phenotype; high levels of these factors are typical of the pathology [67, 69, 77].

These cytokines have different functions: IL-12 promotes further induction of adaptive cellular responses; IL-10 has an immunosuppressive effect and is involved in the induction of regulatory T cells; IL-1β, IL-6, IL- 21, IL-23, and TNF-α exert a proinflammatory effect; TNF-α and iNOS provoke cellular damage [78, 79].

The predominant cytokine profile, as well as the presence of M1 macrophage-producing cells in combination with the effect of autoantigens of the damaged spinal cord, suggests that the population of T lymphocytes involved in the immune response at the initial stage includes Th17 cells whose functional significance during the immediate post-traumatic period of a spinal cord injury has already been proved. The functional role of this subpopulation is closely related to the formation of the balance T helper 17/regulatory T cells (Th17/Treg). Q. Fu et al. [29] described these processes as follows: the Th17/Treg cell balance is regulated by the molecules RORγT and FoxP3, while FoxP3 expression can be inhibited by RORγT expression. As mentioned above, a spinal cord injury is accompanied by the migration of M1 macrophages to the injury site and release of proinflammatory cytokines, including IL-6 and IL-21. This allows T-helpers (CD4^+^ T lymphocytes) to differentiate into CD4^+^IL^-^17A+ Th17, which contribute to the inflammatory response by recruiting neutrophilic granulocytes. In combination with proinflammatory cytokines secreted at the injury site by macrophages, neurons, and neuroglia cells, the products of Th17 and neutrophils greatly exacerbate the inflammation, which is regarded as a quite undesirable aspect of the pathogenesis of post-traumatic changes in the spinal cord.

It should also be emphasized that Th17 induction during the initial phase requires one more cytokine, the transforming growth factor β (TGFβ), which is mainly secreted by Treg cells. The formation of these cells that play an important role in the Th17/Treg balance is mainly mediated by IL-10, which is also secreted by M1 macrophages in relatively small amounts during the initial phase of tissue damage. Like TGFβ, IL-10 also has an immunosuppressive effect, thus limiting the redundancy of the autoimmune inflammatory process after a spinal cord injury [77, 80].

Thus, the innate immune response and T cell-mediated responses that prevail during the immediate pre-traumatic phase of a spinal cord injury should be assessed in a different manner. On the one hand, they aim to eliminate cells in the damaged spinal cord tissue through apoptosis or cytolysis, as well as induce an inflammatory response that enhances neurological dysfunction. On the other hand, these reactions contribute to the elimination of the destroyed cell elements, along with the corresponding autoantigens, injury patterns, and inflammation mediators, and they also involve the mechanisms that regulate inflammatory responses. These conclusions require one not to use a simplified approach to assess the role played by immune processes in a spinal cord injury. They also affect the chosen therapeutic strategy during the immediate post-traumatic period, as one needs to evaluate the balance between the immune mechanisms that prevail in each particular case and exhibit either a protective or pathogenetic action, rather than individual parameters.

## 2. THE IMMUNE AND CYTOKINE PROCESSES ACCOMPANYING THE CHRONIC PHASE OF A SPINAL CORD INJURY

As early as during the immediate post-traumatic phase, a spinal cord injury causes a severe inflammatory response [81] and a strong immune response both within and beyond the injury site [82]; these responses do not tend to resolve. In this case, the interaction takes place between the CNS and the immune system (i.e., the two main systems maintaining homeostasis in the entire body). That is why the process involves not only the response of immune cells in the site of the spinal cord injury but also affects one’s entire immune system [83].

The functions of the immune system change significantly as the immediate post-traumatic phase after the injury progresses to a chronic phase. The loss or dysfunction of vegetative innervation in the lymphatic and endocrine tissues causes immune response disorders that last quite a long time after the initial trauma [84]. The main manifestations of such disorders are immune depression and the autoimmune process [83], although inflammatory reactions also remain pathogenetically significant.

Thus, starting on day 7 after a spinal cord injury, signs of regeneration of the myelin sheath of neurons, accompanied by a biochemically detectable activity of oligodendrocytes and production of the proinflammatory cytokines TNF-α, IL-1β, and IL-6, were observed [85]. Meanwhile, it was noted that the higher the level of proinflammatory cytokines during the chronic phase, the sooner the remission after the spinal cord injury occurs [86].

The fact is that proinflammatory cytokines trigger the activation of astrocytes in the spinal cord [87]. Astrocytes undergo proliferation and acquire one of two phenotypes; astrocytes that have one phenotype and actively secrete a glial fibrillary acidic protein (GFAP), which contributes to neuroregeneration. Contrariwise, astrocytes that have the other phenotype and secrete the glutamine synthase that is involved in the glutamate uptake and slows down neuronal regeneration in the injured spinal cord region. The balance between astrocytes with these two phenotypes determines the efficiency of neuroregeneration [88]. Neurons secrete neuregulin-1 (Nrg-1), which stimulates cell regeneration, contributes to the preservation of the spinal cord white matter, and positively regulates the functions of macrophages, T cells, and B cells. Today, it is even recommended as a medicinal product for patients with spinal cord injury [89].

Although this positive regulation is possible, one should take into account the fact that all the aforementioned processes take place in the CNS; therefore, they can have both local and systemic manifestations.

Systemic changes at the level of cell populations and lymphocyte subpopulations during the chronic phase of a spinal cord injury are mainly related to T cell-mediated adaptive immunity. Thus, it has been demonstrated that the total count of T cells (CD3+) and T helper cell subpopulation (CD3+ CD4^+^) in the blood is reduced, although the count of activated CD4^+^ T cells (HLA-DR+CD4^+^) remains elevated [90]. This is possible if the count of T helper cells in the blood decreases because they migrate to the affected organ.

Regulatory T cells (Tregs) that exhibit suppressive properties are particularly interesting in this case. These cells have a CD3+CD4^+^CD25+CD127lo phenotype; the activated CCR4+HLA-Dr+ fraction being the predominant one. The level of the transforming growth factor β (TGFβ), the main cytokine in these cells, is significantly higher in patients with spinal cord injury, which largely explains the observed immune dysfunction and its sequelae, such as impaired defense against infections and/or persistent chronic inflammation [5, 38].

The deficiency of T-cell-mediated immunity at a systemic level is also accompanied by a significant reduction in NK cell count during the chronic phase of SCI, which eventually often leads to the development of a lethal infection [91].

**Fig. 3:**
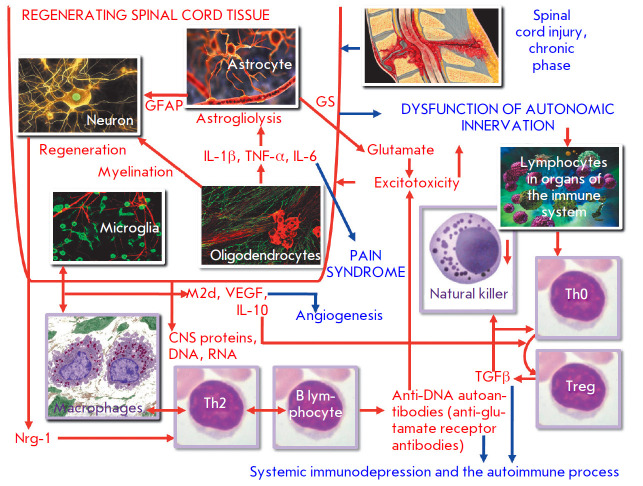
Specific immunopathogenetic characteristics of the chronic post-traumatic phase after SCI

Speaking about one of the key mechanisms of induction of the observed changes, we would like to provide the data obtained by C.J. Ferrante and S.J. Leibovich [77]. They reported that after the immediate tissue damage phase, the macrophage phenotype switched abruptly from M1 to M2, which significantly differs from the typical M2 cells in terms of cytokine secretion. This variety was called the angiogenic M2d phenotype. The main products of M2d macrophage secretion included the vascular endothelial growth factor (VEGF) and IL-10 inducing the formation of regulatory T cells. That is why the angiogenic and immunosuppressive effects are predominant (see *Fig. 3*). Similar transformations also took place for macrophage microglial cells [92].

Special attention should be paid to the autoimmune processes associated with a spinal cord injury. D.P. Ankeny et al. [93] demonstrated that a spinal cord injury and the immunodepression accompanying it cause profound long-lasting changes in the functions of B cells in the peripheral lymphoid tissue (the bone marrow and spleen) and the injured spinal cord; in particular, after differentiation-activated B cells become able to secrete autoantibodies that bind to CNS proteins and nuclear antigens, including DNA and RNA. In patients with systemic lupus erythematosus, anti-DNA antibodies cross-reactively interact with glutamate receptors to cause excitotoxicity [94]. The same phenomenon is observed for the autoantibodies produced in patients after SCI that exhibit similar neurotoxic properties.

After a spinal cord injury, the autoimmunity can also promote CNS re-generation and/or neuroprotection, although there still can be a tendency towards neurotoxicity manifestations. Myelin-reactive T cells exhibit a similar neuroprotective effect in a rat model of SCI [95]. The data on the role played by autoantibodies are rather inconsistent, because the antibodies specific to CNS proteins can promote axonal re-generation and remyelination [96], as well as demyelination, because antimyelin antibodies can be involved in the formation of a “bridge” between myelin of nerve fibers and oligodendrocytes [97]. In any case, despite the ambiguity of the effects and their interpretations, it has been established that B cells infiltrate the injured spinal cord during the chronic phase [93].

The presented analysis demonstrates that interpreting the results is challenging, because it is rather difficult to differentiate between local and systemic effects after a spinal cord injury. In this regard, the possibility of differentiating between the local and systemic manifestations of the immune response opens some prospects. For example, significant changes in the cytokine profile after SCI, especially during the chronic phase, were observed not only in the blood. The Cchanges in the cytokine profile in CSF were even more informative. Thus, A.R. Taylor et al. [98] determined the levels of the IL-2, IL-6, IL-7, IL-8, IL-10, IL- 15, IL-18, granulocyte-macrophage colony-stimulating factor (GM-CSF), interferon-γ (IFNγ), keratinocyte chemoattractant (KC-like protein), IFNγ-inducible protein 10 (IP-10), monocyte chemotactic protein-1 (MCP-1), and tumor necrosis factor α (TNF-α) in the cerebrospinal fluid as a criteria for evaluating the intensity of a chronic inflammation. The concentrations of most cytokines and chemokines in CSF of animals after SCI correlated with injury duration, injury severity at sampling, and the long-term neurological outcome. Thus, the IL-8 level after a spinal cord injury was significantly higher than in the healthy control but showed a negative correlation with injury duration; the levels of colony-stimulating factors and MCP-1 negatively correlated with a long-term positive outcome.

Particular focus is to be directed at the role played by tumor necrosis factor α during the chronic phase after a spinal cord injury. The fact is that the level of brain-derived neurotrophic factor (BDNF) decreases in the hippocampus while increasing in the lateral part of the spinal cord. Deletion within the gene encoding the TNF-α receptor cancels this effect, but the presence of this cytokine restores it. These findings suggest that the various structural synaptic changes in the spinal cord and hippocampal neurons are mediated by overproduction of TNF-α by activated microglial cells, which can be associated with the development of chronic neuropathic pain and memory deficit after a spinal cord injury [99].

IL-1β that reduces the efficiency of the calcium pump function in neurons is also involved in the development of neuropathic pain [100].

Hence, cytokines contribute rather significantly to the pathogenesis of a traumatic disease after a spinal cord injury and are responsible for many of its manifestations. The cytokines can be secreted by the immune cells; however, the neurons of the damaged spinal cord are the main source of these biologically active substances. Therefore, the cytokine profile in patients with SCI plays a special diagnostic and prognostic role. It also characterizes both the immune and neurological status of patients with this pathology.

## CONCLUSIONS

This review of publications focused on the problem of the immune (including cytokine) processes accompanying a spinal cord injury demonstrates that the available data are ambiguous and difficult to interpret.

The complexity of the problem is primarily to do with the fact that both the nervous and immune systems have important regulatory functions in the body and are tightly interrelated, while the mechanisms behind this interrelation are very diverse. Both local and systemic manifestations accompany the neurological and immune changes that occur after a spinal cord injury.

Along with these general aspects, it is important to take into account the phases of local and systemic changes in the central nervous system and the immune processes associated with SCI [101, 102]. Each phase is characterized by its own predominant pathogenetic mechanism, which is initially associated with the response to the injury and aims to eliminate the damaged cells; then, the focus moves towards the inflammatory response aiming to confine the affected area to a minimum. Finally, a transition from local responses to systemic processes takes place during the last stages; the outcome of the pathological process depends on the efficiency of these phases. Each phase is accompanied by its own category of immune response; various cell subpopulations characteristic of innate and adaptive immunity or cytokines, the secretory products of these cells, can act as markers of these types of immune response [103, 104].

A specific feature of cytokines as markers of pathological changes after a spinal cord injury is that they are secreted not only by immune cells, but also by the cells of the damaged spinal cord. The interaction between the nervous and immune systems can be observed using the cytokine profile model, which is both of fundamental interest and diagnostic importance as it allows one to identify the key targets of therapeutic action.

## Abbreviations

BBB: blood–brain barrier
SC: spinal cord
SCI: spinal cord injury
CNS: central nervous system
